# A propos de trois frères atteints d'une arthropathie goutteuse secondaire à un déficit enzymatique en HGPRT

**DOI:** 10.11604/pamj.2014.19.271.3610

**Published:** 2014-11-11

**Authors:** Rim Akrout, Soufiene Baklouti

**Affiliations:** 1 Service de Rhumatologie, CHU Hédi Chaker, Sfax,Tunisie

**Keywords:** Arthropathie goutteuse, déficit enzymatique en HGPRT, flessum irréductible, nodules, gouty arthropathy, HGPRT enzyme deficit, irreducible flexion, nodules

## Image en medicine

Un patient âgé de 23 ans, pécheur, issu de parents non consanguins qui présentait une polyarthrite déformante associant un flessum irréductible des coudes et des genoux avec limitation des amplitudes articulaires. Ces manifestations articulaires étaient associées à des nodules localisés en regard des articulations atteintes (A,B) et au niveau des pavillons des oreilles. La biologie révèle une VS à 60 mm à la première heure, une créatinémie à 91 µmol/l et une uricémie à 1054 µmol/l. La sérologie rhumatoïde était négative. La ponction d'un nodule met en évidence des dépôts d'urate de sodium. Le diagnostic de goutte a été retenu et on a complété par le dosage de l'HGPRT qui a révélé un déficit sévère à 0,2 (valeur usuelle 2,46 + /-0,42) soit 10% d'activité résiduelle. La radiologie a montré des images destructrices avec des macrogéodes au niveau des mains (C,D) et des pieds. Un traitement hypouricémiant a été instauré après traitement de la crise. Son frère ainé âgé de 29 ans, suivi depuis l’âge de 21 ans pour une polyarthrite asymétrique évoluant par crise cédant à la colchicine. Un troisième frère âgé de 21 ans, était en outre suivi dans notre service depuis l’âge de 19 ans pour goutte. Les trois frères présentaient un déficit en HGPRT. La suppression d'aliments riches en bases puriques (abats, charcuterie) a été conseillée et un traitement hypouricémiant était instauré.

**Figure 1:**
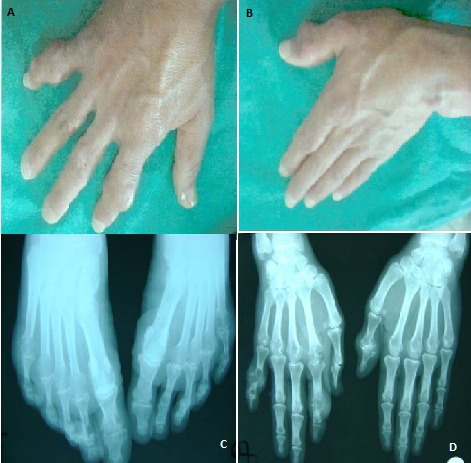
(A,B) tophi goutteux au niveau des mains, (C,D) macrogéodes au niveau des mains et des pieds

